# Economic Evaluation of Enhanced vs Standard Varenicline Treatment for Tobacco Cessation

**DOI:** 10.1001/jamanetworkopen.2024.8727

**Published:** 2024-04-29

**Authors:** Marlon P. Mundt, James H. Stein, Michael C. Fiore, Timothy B. Baker

**Affiliations:** 1 Department of Family Medicine and Community Health, University of Wisconsin School of Medicine and Public Health, Madison; 2 Department of Population Health Sciences, University of Wisconsin School of Medicine and Public Health, Madison; 3 Center for Tobacco Research and Intervention, University of Wisconsin School of Medicine and Public Health, Madison; 4 Division of Cardiovascular Medicine, Department of Medicine, University of Wisconsin School of Medicine and Public Health, Madison

## Abstract

**Importance:**

Smoking is the leading preventable cause of death and illness in the US. Identifying cost-effective smoking cessation treatment may increase the likelihood that health systems deliver such treatment to their patients who smoke.

**Objective:**

To evaluate the cost-effectiveness of standard vs enhanced varenicline use (extended varenicline treatment or varenicline in combination with nicotine replacement therapy) among individuals trying to quit smoking.

**Design, Setting, and Participants:**

This economic evaluation assesses the Quitting Using Intensive Treatments Study (QUITS), which randomized 1251 study participants who smoked into 4 conditions: (1) 12-week varenicline monotherapy (n = 315); (2) 24-week varenicline monotherapy (n = 311); (3) 12-week varenicline combination treatment with nicotine replacement therapy patch (n = 314); or (4) 24-week varenicline combination treatment with nicotine replacement therapy patch (n = 311). Study enrollment occurred in Madison and Milwaukee, Wisconsin, between November 11, 2017, and July 2, 2020. Statistical analysis took place from May to October 2023.

**Main Outcomes and Measures:**

The primary outcome was 7-day point prevalence abstinence (biochemically confirmed with exhaled carbon monoxide level ≤5 ppm) at 52 weeks. The incremental cost-effectiveness ratio (ICER), or cost per additional person who quit smoking, was calculated using decision tree analysis based on abstinence and cost for each arm of the trial.

**Results:**

Of the 1251 participants, mean (SD) age was 49.1 (11.9) years, 675 (54.0%) were women, and 881 (70.4%) completed the 52-week follow-up. Tobacco cessation at 52 weeks was 25.1% (79 of 315) for 12-week monotherapy, 24.4% (76 of 311) for 24-week monotherapy, 23.6% (74 of 314) for 12-week combination therapy, and 25.1% (78 of 311) for 24-week combination therapy, respectively. The total mean (SD) cost was $1175 ($365) for 12-week monotherapy, $1374 ($412) for 12-week combination therapy, $2022 ($813) for 24-week monotherapy, and $2118 ($1058) for 24-week combination therapy. The ICER for 12-week varenicline monotherapy was $4681 per individual who quit smoking and $4579 per quality-adjusted life-year (QALY) added. The ICER for 24-week varenicline combination therapy relative to 12-week monotherapy was $92 000 000 per additional individual who quit smoking and $90 000 000 (95% CI, $15 703 to dominated or more costly and less efficacious) per additional QALY.

**Conclusions and Relevance:**

This economic evaluation of standard vs enhanced varenicline treatment for smoking cessation suggests that 12-week varenicline monotherapy was the most cost-effective treatment option at the commonly cited threshold of $100 000/QALY. This study provides patients, health care professionals, and other stakeholders with increased understanding of the health and economic impact of more intensive varenicline treatment options.

## Key Points

**Question:**

What is the cost-effectiveness of enhanced varenicline treatment (extended varenicline use or varenicline in combination with nicotine replacement therapy) among individuals trying to quit smoking?

**Findings:**

In this economic evaluation of enhanced varenicline treatment for smoking cessation that included 1251 participants, the incremental cost-effectiveness ratio was $4579 per quality-adjusted life-year (QALY) for 12-week varenicline monotherapy. In contrast, 24-week varenicline combination therapy cost $90 000 000 per additional QALY relative to 12-week varenicline monotherapy.

**Meaning:**

This study suggests that standard 12-week varenicline monotherapy is the most cost-effective treatment option for smoking cessation at the commonly cited threshold of $100 000/QALY.

## Teaser

This economic evaluation examines the cost-effectiveness of varenicline monotherapy vs varenicline treatment in combination with patch nicotine replacement therapy for smoking cessation when both types of treatment are offered at standard of care and extended durations.

## Introduction

Smoking is the leading preventable cause of death and illness in the US, responsible for approximately 480 000 deaths annually or 1 in 5 deaths annually.^1^ Evidence-based strategies to aid smoking cessation include behavioral counseling and pharmacotherapy to lower nicotine reinforcement and withdrawal from nicotine. Varenicline treatment for aiding smoking cessation has been shown to be effective for diverse populations of smokers and has been used with different strategies aimed at increasing its effectiveness.^2,3^ Available research presents mixed results about the effectiveness of combination varenicline therapy enhanced with a nicotine replacement therapy (NRT) patch vs varenicline alone.^4,5,6,7^ Although one large study revealed a statistically significant difference in favor of combination varenicline and NRT patch therapy, 2 smaller studies did not show a statistically significant improvement with the combination treatment.^4,5,6^ A meta-analysis of the 3 studies revealed a statistically significant benefit associated with varenicline treatment enhanced with the NRT patch.^7^ In view of conflicting evidence, the American Thoracic Society chose to conditionally recommend varenicline plus the NRT patch rather than varenicline monotherapy for tobacco cessation until future studies explored this issue further.^8^ Most smoking cessation guidelines encourage varenicline monotherapy, NRT patch, bupropion, or a combination therapy with different types of NRT.

Some research shows that increased duration of smoking cessation pharmacotherapy may enhance its effectiveness.^9,10,11^ However, to our knowledge, limited research exists on the benefits associated with extended varenicline treatment. Two smoking relapse prevention studies^12,13^ and a Cochrane meta-analysis^14^ indicate that prolonged duration of varenicline treatment can increase long-term cessation rates from prior treatment among individuals who have quit smoking. There is limited understanding on whether combining varenicline with the NRT patch or prolonging varenicline treatment duration increases smoking cessation rates and/or whether the combination or prolonged varenicline treatments are cost-effective.

To fill this gap in literature, the Quitting Using Intensive Treatments Study (QUITS) randomized clinical trial compared the efficacy of extending varenicline treatment duration and/or offering varenicline treatment in combination with the NRT patch as a means of increasing smoking treatment effectiveness. The primary treatment outcome findings from the QUITS trial were published in a prior report.^15^

This economic evaluation examines the cost-effectiveness of varenicline monotherapy vs varenicline treatment in combination with the NRT patch when both types of treatment are offered at standard of care and extended durations. This economic analysis informs patients, health care professionals, policy makers, and other stakeholders about the health and economic outcomes of standard varenicline monotherapy vs combination and extended varenicline treatment strategies.

## Methods

This study is an economic evaluation alongside the QUITS randomized clinical trial.^15^ The analysis was conducted from a health care system perspective based on QUITS costs and effectiveness over a 12-month follow-up. The study followed the Consolidated Health Economic Evaluation Reporting Standards (CHEERS) 2022 reporting guideline. The study participants were individuals who smoked daily and who expressed an interest in quitting smoking by responding to study recruitment for the smoking cessation intervention trial. The QUITS randomized clinical trial was approved by the University of Wisconsin Health Sciences Institutional Review Board. All participants provided written informed consent.

Available evidence in the literature shows that race is associated with tobacco cessation treatment outcomes.^16,17,18^ Race and ethnicity were self-reported by participants using fixed choice responses. Study group enrollment was stratified by race and ethnicity to prevent chance association of this variable with treatment assignment.

### QUITS Study Design Overview

Recruitment occurred via community outreach (eg, social networking sites). Interested respondents were called for eligibility assessment, consent, baseline assessments, and randomization. Study enrollment occurred at 1 research clinic in Madison, Wisconsin, and 1 research clinic in Milwaukee, Wisconsin, between November 11, 2017, and July 9, 2020.

Inclusion criteria included English language proficiency, smoking 5 or more cigarettes per day during the past 6 months, exhaled carbon monoxide level of 5 ppm or greater, age 18 years or older, a desire to quit smoking, not currently engaged in smoking cessation treatment, no use of other tobacco products (pipe tobacco, cigars, snuff, e-cigarettes, or chewing tobacco) within the past 30 days, telephone access, willingness and ability to use both the NRT patch and varenicline, ability to attend clinic visits for the next 12 months, not currently pregnant, and agreement to use an acceptable birth control method. More details are provided in the report by Baker et al.^15^

### Randomization

Participants were randomized to medication condition (varenicline monotherapy vs combination varenicline and NRT patch) and treatment duration (12 weeks vs 24 weeks) via a database that used stratified permuted block randomization. SAS Proc Plan, version 9.4 (SAS Institute Inc) was used to stratify by site (Madison or Milwaukee), sex, race and ethnicity (Asian, Black or African American, Native American or Alaska Native, Native Hawaiian or Other Pacific Islander, or non-Hispanic White [hereinafter, White], other [including those who chose other or did not answer], or >1 race; groups were dichotimized as White and all other groups), and smoking heaviness (5-15 [low] or ≥16 [high] cigarettes/d), with a fixed block size of 4 based on the 4 unique treatment groups (in random order within each block). Participants were randomized by block, with one randomization block being White individuals and the other randomization block being individuals of all other races and ethnicities; the literature indicated that smoking cessation rates may vary by race and ethnicity between White individuals and those of all other race and ethnicity groups. The double-blind treatment assignment meant that all participants took pills and wore NRT patches for the same treatment length.

### Interventions

Study medications were dispensed 1 week before target quit date and at the target quit date and were mailed to participants at week 10 after baseline. The 4 treatment conditions were created by the 2 × 2 factorial design: (1) varenicline monotherapy for 12 weeks: active varenicline from target quit date to week 13 and placebo varenicline from week 14 to week 25 plus placebo patch from baseline to week 25; (2) varenicline plus NRT patch for 12 weeks: active varenicline from target quit date to week 13 and placebo varenicline from week 14 to week 25 plus active NRT patch from baseline to week 13 and placebo patch from week 14 to week 25; (3) varenicline monotherapy for 24 weeks: active varenicline from target quit date to week 25 and placebo patch from baseline to week 25; and (4) varenicline plus NRT patch for 24 weeks: active varenicline from target quit date to week 25 plus active NRT patch from baseline to week 25.

To mask treatment assignment, all participants were given 24 weeks of varenicline pills and 26 weeks of NRT patches, with some medications being active and some placebo. Varenicline treatment started with one 0.5-mg varenicline pill for 3 days, two 0.5-mg pills for 4 days, and two 1-mg pills thereafter (to either week 13 or week 25). Active NRT patch use started at baseline and involved use of one 14-mg patch/d until either 13 weeks or 26 weeks after the target quit day. Placebo products had the same appearance and use instructions as the respective active products.

### Cessation Counseling

All participants were scheduled for six 15-minute counseling sessions, with 3 sessions during face-to-face visits at study entry, week 2, and week 4 plus 3 more sessions by telephone call at weeks 1, 6, and 10. Counseling focused on instructions for medication use, support, coping skills, and motivation to quit. A manual was created to standardize the counseling, and the audio during the sessions was recorded for quality assurance assessment and feedback.

### Assessments

Questionnaires assessing smoking history, nicotine use, nicotine dependence, and negative affect were conducted at study entry. Participants were contacted 52 weeks after the target quit day for a telephone assessment of smoking status, use of other nicotine products or cessation aids, medication use, and adverse events. Participants claiming abstinence from smoking at 52 weeks were invited for in-person biochemical verification.

Medication adherence was assessed by self-report for the 7 days prior to each study contact from week 1 to week 24 (2 visits and 6 telephone calls). Adherence to varenicline treatment was defined post hoc as taking 1 or 2 pills per day for 6 days or longer; adherence to treatment with the NRT patch was defined as using 1 patch per day for 6 days or longer.

### Outcome Measures

The primary outcome was self-reported 7-day point prevalence abstinence (biochemically confirmed with exhaled carbon monoxide level ≤5 ppm) at 52 weeks after the target quit day. COVID-19 restrictions prevented biochemical confirmation of abstinence for some participants from March to July 2020 (5.9% of the total sample [74 of 1251]).

### Cost Measures

Intervention costs included salaries, benefits, and resources for tobacco cessation counseling and tobacco cessation medications associated with the intervention. Medicare reimbursement rates of $34 per counseling session (*Current Procedural Terminology* codes 99406 and 99407), $392 per 28-day prescription of varenicline, and $84 per 28-day prescription of the NRT patch were applied to treatments received by the study participants.^19,20^ All costs were reported in 2021 dollars.

Although all study participants were enrolled to receive 6 tobacco cessation counseling sessions and all were provided the full duration of varenicline and NRT patch, costs were calculated based on the actual treatments used by the participants. To simulate real-world prescribing of varenicline, costs were based on a 30-day starter pack of varenicline for all participants; if the participants reported being adherent to treatment, they were provided subsequent 28-day supplies until treatment conclusion. Similarly, NRT patch costs were based on active NRT patch group participants receiving the initial 14-day supply of patches and subsequent supplies of patches if the participant remained adherent to NRT patch treatment. If a participant reported treatment discontinuation, the cost of 1 additional month of medication was added, but costs were assumed to be zero thereafter. Costs for placebo medications were considered research costs and were not included in the evaluation.

### Statistical Analysis

Statistical analysis took place from May to October 2023. Cost-effectiveness was estimated by the incremental cost-effectiveness ratio^21^ (ICER), calculated as follows:
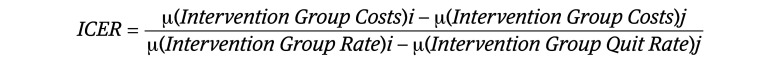
for comparing treatment condition *i* with treatment condition *j*.

**Figure 1.**
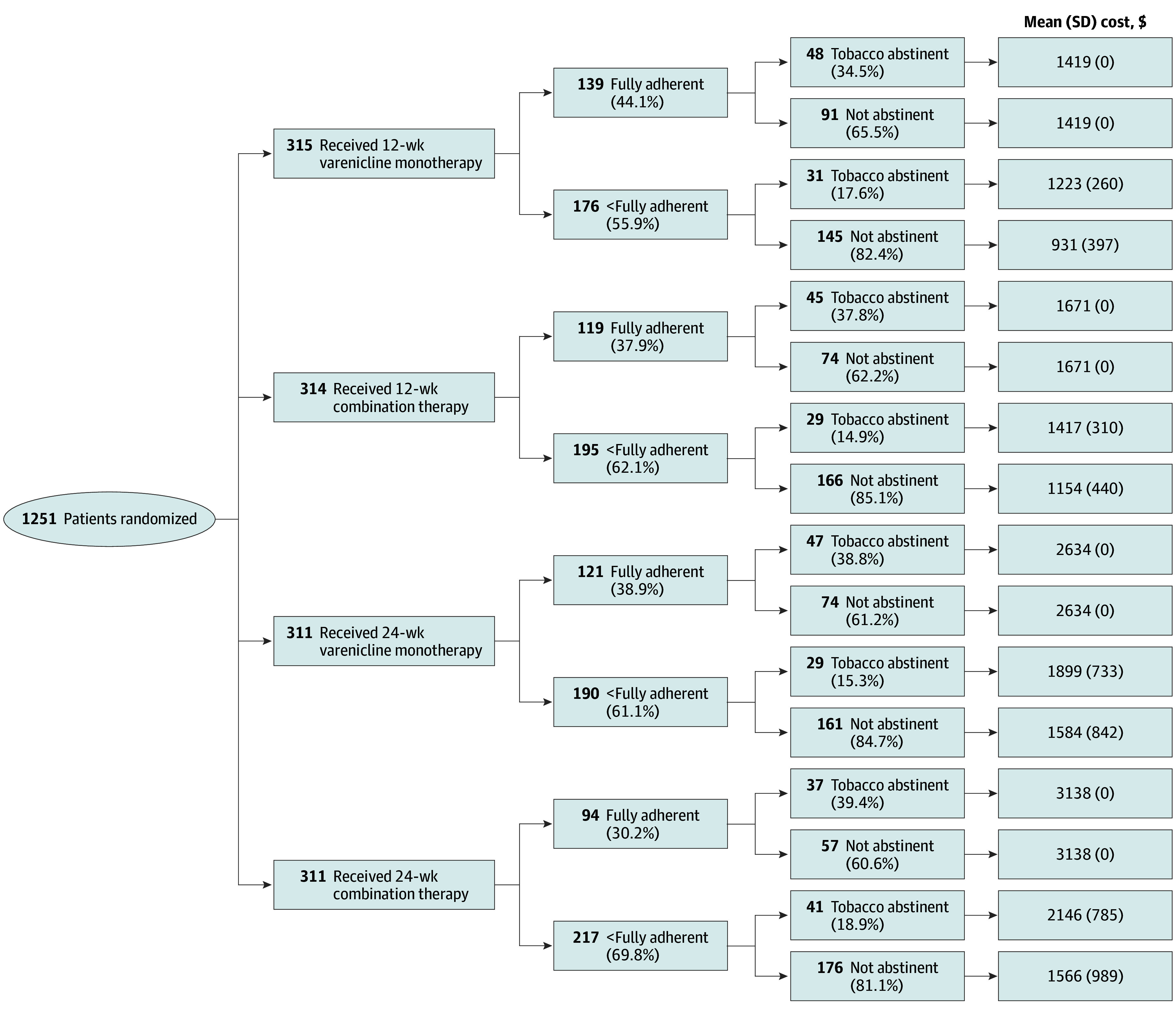
Cost-Effectiveness Decision Tree

To evaluate the ICER for each additional individual who quit smoking, participants in the trial were determined to be either tobacco abstinent (cessation = 1) or returned to smoking (cessation = 0) at the 52-week follow-up. Individual costs included the number of cessation counseling sessions completed and cessation medications used. Mean cessation rate and mean cost were computed for each randomization-outcome group based on whether the participants had completed the assigned treatment regimen, resulting in 8 treatment-outcome combinations (see Figure 1 for the pathway probabilities).

A combination of probabilistic sensitivity analysis and Monte Carlo–based nonparametric bootstrap analysis was used to account for uncertainty in parameters and to determine 95% CIs for the ICER per additional individual who quit smoking.^22,23,24^ The ICER per additional individual who quit smoking was calculated by drawing repeated random samples with replacement (N = 1000) from the observed distributions for cessation and health care costs to produce bootstrap estimates of the 95% CI.

In addition, the ICER per additional participant who quit smoking was translated into a cost per additional quality-adjusted life-year (QALY) gained using conversion factors from Stapleton and West.^25^ Stapleton and West^25^ estimate life-years gained from stopping smoking at various ages, probabilities of relapse back to smoking, and discounting of future benefits (ie, life-years gained are accrued in the future, at the end of life) so that quit rate differentials can be converted into QALYs. We used Table 3, Section B from Stapleton and West^25^^(p468)^ to convert incremental cost and incremental quit rates to a cost per QALY using linear interpolation to estimate between table cells. A discount rate of 3% was used for the QALY conversion.

The χ^2^ test was conducted to compare withdrawal rates and loss-to-follow-up rates across randomization groups. All *P* values were from 2-sided tests, and results were deemed statistically significant at *P* ≤ .05. Statistical tests of cost-effectiveness were conducted by determining if the bootstrap 95% CIs contained the cost-effectiveness threshold of $100 000/QALY. If the entire 95% CI for cost-effectiveness lay below the $100 000/QALY threshold, we concluded that the treatment was cost-effective at the *P* = .05 level. Subgroup analyses included differences in cost-effectiveness by age, race and ethnicity, educational level, prior use of varenicline, tobacco dependence, motivation to quit smoking, and confidence in quitting smoking.

## Results

Among the 1251 patients in the study, mean (SD) age was 49.1 (11.9) years, 675 (54.0%) were women, 576 (46.0%) were men, 287 (22.9%) were Black or African American, and 867 (69.3%) were White (Table 1). A total of 751 participants (60.0%) completed the assigned treatment, 966 (77.2%) completed 12-month follow-up, and 881 (70.4%) completed 52-week follow-up. A total of 117 enrolled participants (9.4%) withdrew from the study and 168 (13.4%) were lost to follow-up. There were no significant differences in withdrawal or loss to follow-up by study group. Participants smoked a mean (SD) of 16.0 (7.5) cigarettes/d prior to the intervention (Table 1).

**Table 1.** Demographic and Smoking-Related Variables for Study Participants

Variable	Patients, No. (%)				
	All (N = 1251)	Varenicline monotherapy		Combination therapy^a^	
		12 wk (n = 315)	24 wk (n = 311)	12 wk (n = 314)	24 wk (n = 311)
Age, mean (SD), y	49.1 (11.9)	48.9 (12.4)	48.9 (12.3)	48.6 (11.4)	49.9 (11.5)
Sex					
Male	576 (46.0)	144 (45.7)	144 (46.3)	143 (45.5)	145 (46.6)
Female	675 (54.0)	171 (54.3)	167 (53.7)	171 (54.5)	166 (53.4)
Race					
Asian	8 (0.6)	2 (0.6)	1 (0.3)	2 (0.6)	3 (1.0)
Black or African American	287 (22.9)	70 (22.2)	78 (25.1)	71 (22.6)	68 (21.9)
Native American or Alaska Native	14 (1.1)	5 (1.6)	2 (0.6)	3 (1.0)	4 (1.3)
Native Hawaiian or Other Pacific Islander	3 (0.2)	1 (0.3)	2 (0.6)	0	0
White	867 (69.3)	219 (69.5)	214 (68.8)	221 (70.4)	213 (68.5)
Other^b^	44 (3.5)	12 (3.8)	6 (1.9)	12 (3.8)	14 (4.5)
>1 Race category	27 (2.2)	6 (1.9)	7 (2.3)	5 (1.6)	9 (2.9)
Ethnicity					
Hispanic	41 (3.3)	10 (3.2)	5 (1.6)	14 (4.5)	12 (3.9)
No. of cigarettes/d, mean (SD)	16.0 (7.5)	15.9 (7.6)	16.2 (7.4)	16.0 (7.3)	16.0 (7.7)
Motivation to quit, mean (SD)^c^	6.4 (0.9)	6.4 (0.9)	6.4 (0.8)	6.4 (0.9)	6.4 (0.9)
Confidence in quitting, mean (SD)^d^	5.5 (1.3)	5.5 (1.4)	5.5 (1.3)	5.5 (1.3)	5.5 (1.3)

^a^ Combination therapy consisted of varenicline treatment in combination with nicotine replacement therapy patch.

^b^ Included those who chose the option “Other” as their racial category and those who did not provide any answer to the race question.

^c^ Measured on a scale of 1 to 7; 1 indicates not at all motivated to quit smoking and 7 indicates extremely motivated to quit smoking.

^d^ Measured on a scale of 1 to 7; 1 indicates not confident in quitting smoking and 7 indicates extremely confident in quitting smoking.

### Intervention Effectiveness and Costs

Tobacco cessation rates at the 52-week follow-up were 25.1% (79 of 315 participants) for 12-week monotherapy, 24.4% (76 of 311) for 24-week monotherapy, 23.6% (74 of 314) for 12-week combination therapy, and 25.1% (78 of 311) for 24-week combination therapy. The mean (SD) intervention cost was $1175 ($365)/person for 12-week monotherapy, $1374 ($412)/person for 12-week combination therapy, $2022 ($813)/person for 24-week monotherapy, and $2118 ($1058)/person for 24-week combination therapy treatment (Table 2). In contrast, maximum costs per individual were $1419 for 12-week monotherapy, $1671 for 12-week combination therapy, $2634 for 24-week monotherapy, and $3138 for 24-week combination therapy treatment if the participant adhered completely to the entire treatment regimen. Adherence to the assigned treatment varied by treatment group, with adherence rates highest in the 12-week varenicline monotherapy group (44.1% [139 of 315] fully adherent) and lowest in the 24-week combination therapy group (30.2% [94 of 311] fully adherent). As seen in the decision tree (Figure 1), mean costs were greater across all 4 groups among those who were tobacco abstinent at 52 weeks compared with those who were nonabstinent, indicating a positive association (*r* = 0.27 [95% CI, 0.22-0.32]) between treatment adherence and smoking cessation rates when controlling for treatment group assignment.

**Table 2.** Costs and Efficacy of Tobacco Cessation Treatment Regimens

Outcome	12-wk Treatment group		24-wk Treatment group	
	Varenicline monotherapy (n = 315)	Combination therapy (n = 314)^a^	Varenicline monotherapy (n = 311)	Combination therapy (n = 311)^a^
Participants fully adherent to treatment regimen, No. (%)	139 (44.1)	119 (37.9)	121 (38.9)	94 (30.2)
Maximum cost per individual if fully adherent to treatment, $	1419	1671	2634	3138
Cost per randomized individual, mean (SD), $	1175 (365)	1374 (412)	2022 (813)	2118 (1058)
52-wk Smoking abstinent, No. (%)	79 (25.1)	74 (23.6)	76 (24.4)	78 (25.1)

^a^ Combination therapy consisted of varenicline treatment in combination with nicotine replacement therapy patch.

### Incremental Cost-Effectiveness Ratio

**Figure 2.**
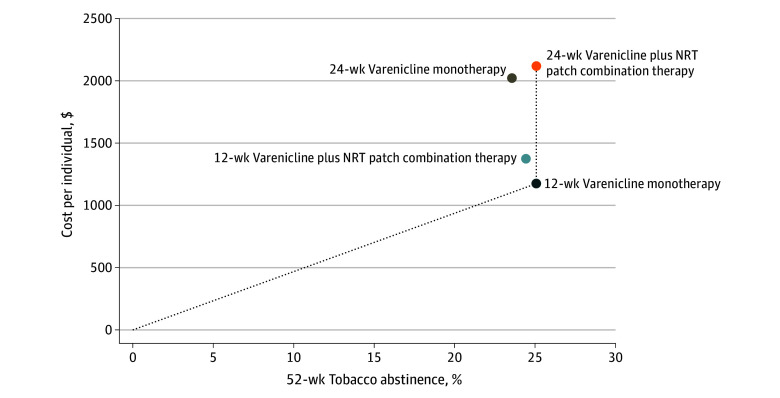
Cost-Effectiveness Plane The dotted lines represent the cost-effectiveness frontier. All points above and to the left of the cost-effectiveness frontier are dominated (more costly and less efficacious) by a treatment option on the frontier. NRT indicates nicotine replacement therapy.

As seen in Figure 2, the plot of the cost-effectiveness plane indicated that 12-week varenicline monotherapy and 24-week varenicline combination therapy lay on the cost-effectiveness frontier. The 2 other treatment combinations, 24-week varenicline monotherapy and 12-week varenicline combination therapy, were dominated (ie, another treatment combination was both cheaper and more effective). The ICER for 12-week varenicline monotherapy was $4681 per individual who quit smoking and $4579/QALY added. On average, 12-week varenicline combination therapy cost $199 more than 12-week monotherapy but was 0.7% less efficacious, hence dominated. Similarly, 24-week varenicline monotherapy cost $847 more than 12-week monotherapy on average but was 1.5% less efficacious, thus also dominated. The slope of the cost-effectiveness curve from 12-week monotherapy to 24-week combination therapy was $92 000 000 per additional individual who quit smoking or $90 000 000 (95% CI, $15 703 to dominated) per additional QALY. The slope of the cost-effectiveness curve between 12-week varenicline monotherapy and 24-week varenicline combination therapy rests above the $100 000/QALY cost-effectiveness threshold.

Incorporating variability in the sensitivity analysis, the results indicate that at the threshold of $100 000/QALY, 47.1% of bootstrap simulations (471 of 1000) yielded 12-week monotherapy as the most cost-effective treatment. In 22.8% of simulations (228 of 1000), 24-week combination therapy was the most cost-effective at the $100 000/QALY threshold.

### Subgroup Analyses

Subgroup analyses by age, sex, race and ethnicity, educational level, number of years smoking, prior use of varenicline, tobacco dependence, motivation to quit smoking, and confidence in quitting smoking did not reveal any subgroups for which the ICER was statistically significant at the $100 000/QALY level. For female, older, White, and more highly educated individuals and for individuals who had used varenicline in a prior smoking cessation attempt, 24-week combination therapy (ie, the most intensive treatment) yielded higher cessation rates than did the less intensive treatment. However, even in the best case for more intensive treatment (age ≥50 years, for whom 24-week combination therapy cessation rates exceeded 12-week monotherapy by 29.9% to 26.7%), the ICER per additional QALY was $33 751/QALY (95% CI, $10 848-$184 982/QALY), with the 95% CI overlapping the $100 000/QALY threshold.

## Discussion

Our study demonstrated that among adults who smoked 5 or more cigarettes per day and who were interested in quitting smoking, 12-week varenicline monotherapy (condition 1) was the most cost-effective pharmacotherapy treatment (ie, ICER = $4681 per additional individual who quit smoking and $4579/QALY). More intense varenicline protocols, which combined varenicline therapy with the NRT patch or that prolonged treatment exposure to varenicline from 12 weeks to 24 weeks, were found not to be cost-effective options at the threshold recommended by the literature of $100 000/QALY in contrast to standard 12-week varenicline monotherapy.^26^ No cost-effective increases in smoking abstinence or projected health benefits were revealed when the QUITS condition 1 participants undergoing 12 weeks of varenicline monotherapy were juxtaposed with each of the other 3 treatment condition groups side by side. Furthermore, increased treatment intensity without commensurate increases in effectiveness can meaningfully increase the ICERs associated with treatment (≤$94 000 000 in the present study). In addition, adherence rates decreased with increased treatment intensity. In sum, our economic evaluation did not offer evidence that would support combining varenicline use with the NRT patch or extending exposure to varenicline therapy.

This cost-effectiveness analysis provides evidence to policy makers and stakeholders on the economic impact of the various intensive varenicline treatment strategies with similar clinical significance and informs their decisions on how limited health care resources could be leveraged to improve patient health outcomes. The positive outcomes seen with modified varenicline pharmacotherapy in prior studies,^7,14^ involving either combination therapy or prolonged varenicline monotherapy, were not replicated in the QUITS trial. Moreover, this pattern of findings was largely invariant across different patient subgroups (ie, age, sex, educational level, and prior varenicline use).

### Limitations

This study has several limitations. First, COVID-19 restrictions from March to July 2020 made it impossible for some participants (6% of the total sample) to biochemically verify their self-report of abstinence at 52 weeks. However, the findings were not meaningfully altered when these individuals were treated as smoking or missing at follow-up for the analysis. Second, medication use decreased over the course of the study. Suboptimal adherence to a medication regimen is a known factor in clinical pharmacotherapy use.^27^ Third, in light of 13.4% of the sample being lost to follow-up at the 52-week follow-up and 9.4% of participants withdrawing from the study, data loss might have decreased the accuracy of the effect sizes observed in the trial and possibly diminished the estimation capacity to detect cost-effectiveness associated with enhanced varenicline treatment.

## Conclusions

In this economic evaluation of standard vs enhanced varenicline treatment for smoking cessation, standard of care varenicline monotherapy was the most cost-effective smoking cessation treatment protocol. Extended varenicline treatment duration and/or the combined use of varenicline with the NRT patch were shown not to be cost-effective at the commonly cited $100 000/QALY threshold used in cost-effectiveness studies. The results also underscore the importance of supplementing the effectiveness evaluation with health economic analyses, as these can provide important complementary information on the relative values of different treatments.

## Notes

Supplement.
Data Sharing Statement
