# Supplementary material for: Economic Evaluation of Enhanced vs Standard Varenicline Treatment for Tobacco Cessation
Source: JAMA Netw Open. 2024 Apr 29;7(4):e248727. doi: 10.1001/jamanetworkopen.2024.8727 (PMC11059041; doi:10.1001/jamanetworkopen.2024.8727)
Supplement: Supplement. — Data Sharing Statement [file jamanetwopen-e248727-s001.pdf]

Data Sharing Statement

Mundt. Economic Evaluation of Enhanced vs Standard Varenicline Treatment for Tobacco Cessation. *JAMA Netw Open*. Published April 29, 2024.
doi:10.1001/jamanetworkopen.2024.8727

Data
Data available: Yes
Data types: Deidentified participant data, Data dictionary
How to access data: tbb@ctri.medicine.wisc.edu
When available: With publication

Supporting Documents
Document types: None

Additional Information
Who can access the data: Researchers whose data use is approved
Types of analyses: meta-analyses, investigation of specific questions judged to be worthwhile by study authors
Mechanisms of data availability: Via request to the authors and with approval by IRB and approval of the University of Wisconsin Center for Tobacco Research and Intervention leadership and trial PI and with a signed access agreement
